# An important reference book for research on barriers between blood and the CNS

**DOI:** 10.1186/1743-8454-2-1

**Published:** 2005-06-12

**Authors:** Hazel C Jones

**Affiliations:** 1 Department of Pharmacology, University of Florida, Gainesville, FL 32610, USA; 2 Gagle Brook House, Chesterton, Bicester, Oxon OX26 1UF, United Kingdom

## 

The health of the central nervous system (CNS) is a subject that impacts on all people and especially on those who have the misfortune to be afflicted by CNS injury or disease. The cerebrovascular barriers at the CNS capillaries and the choroid plexus play a vital role in maintaining the microenvironment of the cells in the brain and spinal cord. The stated aim of this book is to act as a stimulus for research directed towards minimizing the effects of CNS stress, injuries and insults. With seven sections that include 27 chapters from 59 contributors and 605 pages in total, this book covers many topics including: basic aspects of the barriers, *in vitro *models, physiological transport mechanisms, neurochemical mediators, stress situations, changes in disease conditions and clinical aspects. Each chapter consists of a review, together with up-to-date original data. The chapters are laid out clearly in a consistent format with informative illustrations.

This is an important wide-ranging book aimed at researchers in neuroscience, drug discovery and development, and neuropathology. Although large, it contains a wealth of information on contemporary research with an impressive 29-page index from *arachadonic acid *to *zonula occludens proteins*. The bulk of the text deals with research relating to the cerebrovascular barriers in the brain. However, in contrast with past books on the blood-brain barriers, this one contains several chapters concerned with the blood-spinal cord barrier. Considering the volume of research on spinal cord injury in recent decades, this serves as a useful source of information and a reminder that barrier function is important throughout the CNS. Furthermore, it reinforces the probability that information obtained from research on barriers in the brain can be applied to the therapeutic treatment of spinal cord injury.

One rather disappointing aspect of this otherwise excellent book is the small amount of space devoted to the choroid plexus and the cerebrospinal fluid (CSF). This is the alternative route to the brain, the blood-CSF barrier with somewhat different properties to the blood-brain barrier. Only two chapters contribute to this very important aspect of brain homeostasis and to the potential for manipulation of the CSF for diagnostic and therapeutic purposes.

Overall, 'Blood-Spinal Cord and Brain Barriers in Health and Disease' is a contemporary and very informative volume that should be a ready source of reference for all researchers and clinicians concerned with the CNS in health and disease.

## Competing interests

The author(s) declare that they have no competing interests.

## Authors' contributions

Sole author

